# Role of BAF60a/BAF60c in chromatin remodeling and hepatic lipid metabolism

**DOI:** 10.1186/s12986-016-0090-1

**Published:** 2016-04-27

**Authors:** Ping Zhang, Lulu Li, Zhengxi Bao, Feiruo Huang

**Affiliations:** Department of Animal Nutrition and Feed Science, College of Animal Science and Technology, Huazhong Agricultural University, Wuhan, 430070 China

**Keywords:** SWI/SNF complex, Chromatin remodeling, BAF60a, BAF60c, Lipid metabolism, Hepatocyte heterogeneity

## Abstract

The switching defective/sucrose non-fermenting (SWI/SNF) complexes play an important role in hepatic lipid metabolism regulating both transcriptional activation and repression. BAF60a is a core subunit of the SWI/SNF chromatin-remodeling complexes that activates the transcription of fatty acid oxidation genes during fasting/glucagon. BAF60c, another subunit of SWI/SNF complexes, is recruited to form the lipoBAF complex that activates lipogenic genes, promoting lipogenesis and increasing the triglyceride level in response to feeding/insulin. Interestingly, hepatocytes located in the periportal and perivenous zones of the liver display a remarkable heterogeneity in the activity of various enzymes, metabolic functions and gene expression. Especially, fatty-acid oxidation was shown to be mostly periportal, whereas lipogenesis was mostly perivenous. Therefore, the present review highlights the role of of SWI/SNF regulating lipid metabolism under nutritional and hormonal control, which may be associated with hepatocyte heterogeneity.

## Background

Understanding the regulation of hepatic lipid metabolism is critical as this metabolic disorder is often linked to chronic pathological conditions such as fatty liver, obesity, diabetes and cardiovascular disease [1, 2]. Ectopic accumulation of lipid in the liver is an early pathogenic event in the development of nonalcoholic steatohepatitis, characterized by chronic inflammation and liver damage [3, 4]. In recent years, liver lipid metabolism disorders leading to metabolic syndrome are receiving considerable attention. It is well known that lipid metabolism is influenced by several regulatory factors. In this regard, recent studies have shown that the relationship between the upstream stimulatory factor (USF) and the sterol regulatory element-binding protein 1c (SREBP-1c), are two transcription factors shown to be crucial for regulation of lipogenesis [5, 6]. Furthermore, the peroxisome proliferator-activated receptor γ co-activator protein-1α (PGC-1α) was found to play an important role in the regulation of fatty acid β-oxidation (FAO) by interacting with the peroxisome proliferatoractivated receptor-α (PPARα) [7–9].

Other studies have shown a remarkable heterogeneity of periportal- and perivenous hepatocytes along the porto-central axis with respect to ultrastructure and enzyme activities resulting in different cellular functions within different zones of the liver lobule [10, 11]. However, the mechanism underlying zonation was poorly understood in spite of a number of hypotheses that have been proposed. Interestingly, lipid metabolism, with fatty acid oxidation preferentially occurs in the periportal area, whereas lipogenesis takes place predominantly in the perivenous zone [11, 12]. The perivenous hepatocytes have a higher capacity for the *de novo* synthesis of fatty acids; in which the activities of lipid metabolism such as ATP citrate lyase (ACL) [13], acetyl-CoA carboxylase (ACC) [14], and fatty acid synthase (FAS) [15, 16] are higher than that in periportal hepatocytes.

The SWI/SNF chromatin-remodeling complex is known to be involved in the regulation of lipid metabolism [17, 18]. BAF60a and BAF60c, two subunits of the SWI/SNF chromatin-remodeling complexes, are important for maintaining hepatic lipid metabolism. Studies have identified BAF60a and PPARα interacts with PGC-1α for the formation of a transcriptional complex to transcriptional activation of fatty acid oxidation genes during fasting [17]. In contrast, BAF60c recruitment through USF-1 in response to feeding/insulin is specific to lipogenic genes [18]. In this review, we have discussed that under the control of nutritional and hormonal signals, the regulation of hepatic lipid metabolism by SWI/SNF may be involved in the heterogeneity of hepatocytes.

## BAF60a/BAF60c their effects on chromatin-remodeling

### SWI/SNF chromatin-remodeling complexes

**Fig. 1:**
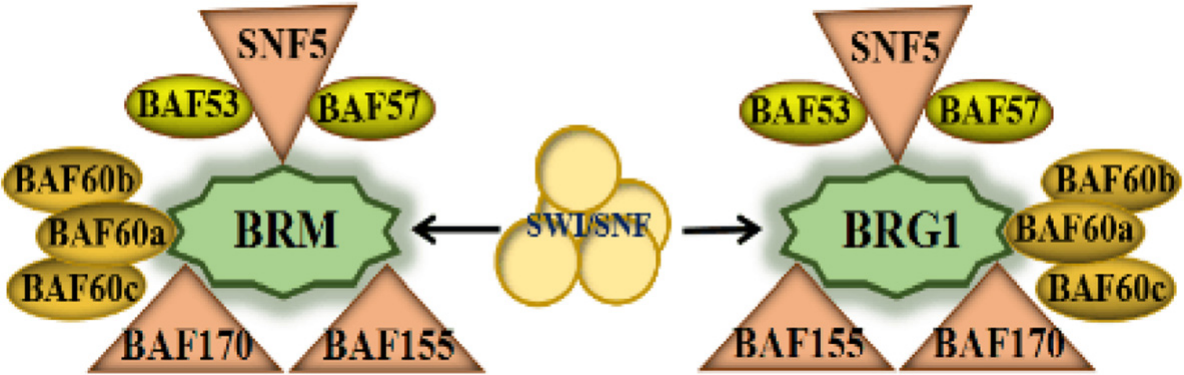
Components of the SWI/SNF complex. SWI/SNF complexes consist of a single ATPase (BRM or BRG1) core subunits (BAF155, BAF170, and SNF5) and accessory subunits (BAF60a, b, or c; BAF57; BAF53)

The components of the switching defective/sucrose non-fermenting (SWI/SNF) chromatin-remodeling complex were initially identified in screens for genes that regulate mating-type switching and sucrose non-fermenting phenotypes in yeasts [19–21]. The SWI/SNF complex components (Fig. 1) contain one of two catalytic ATPases subunits, BRM (brahma, also known as SMARCA2) and BRG1 (brahma-related gene 1, also known as SMARCA4). Each ATPase has 10 to 12 proteins known as BAFs (BRG1- or BRM-associated factors) consisting of core and accessory subunits. The SWI/SNF core subunits include BAF155, BAF170, and SNF5 (also referred to as SMARCB1, BAF47, or INI1). The structure of this multiprotein complex was constructed by superimposing the predicted binary interactions between BAF155 and BRG1, BRG1 and SNF5, and SNF5 and BAF170 [22]. BAF155 maintains a scaffolding-like function, and can influence both stability and assembly of other SWI/SNF subunits [23]. SNF5 has a mediating function in recruiting transcription factors to the SWI/SNF complex. The function of BAF170, which shares homology with BAF155 is less well understood, but it may also control the levels of other SWI/SNF subunits. Thus, the core SWI/SNF subunits encompass widely distinct biochemical and functional activities. The accessory subunits BAF60 proteins have been shown to interact with transcription factors, including nuclear receptors, the AP-1 complex, and others, and are thought to bridge interactions between these transcription factors and BAF complexes [24]. The BAF60 family includes: BAF60a, BAF60b, and BAF60c. They show different expression patterns among tissues and interact with different combinations of nuclear receptors as mediators [25]. The SWI/SNF family of chromatin-remodeling complexes are master regulators of transcription factor action and resultant gene expression programs [26–28].

The SWI/SNF complex is an ATP-dependent chromatin-remodeling complex, which uses the energy of ATP hydrolysis to alter the location or conformation of nucleosomes [29]. Other nonredundant chromatin remodeling complexes endowed with ATPase activity include the ISWI (imitation SWI), CHD (chromodomain and helicase-like domain), and INO80 (inositol requiring 80) families of remodelers [30]. The SWI/SNF complexes have been found to be implicated in the regulation of diverse biological processes, including embryogenesis, cell cycle, differentiation, and tumorigenesis [31, 32]. The SWI/SNF chromatin-remodeling complexes utilize either BRG1 or BRM as alternative catalytic subunits to regulate gene expression [33]. The BRG1 protein can be found assembled with transcription factors and histone-modifying enzyme complexes to activate or repress nuclear processes including transcription, elongation and DNA replication [34]. The combinatorial association of SWI/SNF subunits appears to generate a functional diversity that might be critical for controlling finely selective and cell type-specific developmental and regeneration decisions [35]. The perturbation of SWI/SNF chromatin remodeling complexes is an emerging theme in cancer initiation and progression [36]. Several reports have shown mutations and/or loss of BRG1 in human cancer cell lines and primary tumors [37, 38]. Supporting a role in cancer initiation, loss of heterozygosity of the region surrounding BRG occurs with significant frequency in human adenocarcinomas. Studies have also found that the SWI/SNF complexes regulate cardiovascular development and lipid homeostasis [39, 40]. Overall, the composition of SWI/SNF complexes, which varies by cell type, differentiation stage, and metabolism, might alter the functional interactions with other chromatin modifiers and transcription factors, dictating the specificity for target genes.

### BAF60a function in chromatin-remodeling

In mammals, the BAF complex is a multi-subunit complex that alters the nucleosomes, and is often considered the last step required for transcriptional activation. Within BAF complexes, there are BAF60s proteins that are thought to form a recruitment bridge between DNA-binding transcription factors and other BAF subunits [17]. BAF60a was initially identified as a determinant of the transactivation potential of Fos/Jun dimers to induce the endogenous AP-1-regulated genes such as collagenase and c-met [41]. BAF60a is a subunit of the BAF60 family, regulating nucleosome and chromatin structures through ATP hydrolysis [25]. BAF60a mRNA is present in several tissues, including brain, skeletal muscle, and liver. BAF60a, through Nand C-terminal domains, interlaces BRG1 with BAF170 and BAF155, which forms the core-remodeling complex. It is recruited to the promoter region of a target gene by the specific transcription factor and remodels nearby nucleosomes to facilitate or repress transcription [42]. Studies have also demonstrated [41, 43] that BAF60a recruit SWI/SNF complexes to regulate metabolic gene programs in the liver and skeletal muscle. BAF60a has been linked to lung cancer risk, binds to p53 and is necessary for steroid receptor function [44]. This suggests a pleiotropic role of BAF60a in cellular and organismal biology by integrating endocrine, metabolic, and circadian signals [45]. BAF60 associates with BRG1/BAF190, potentially functioning as a bridge between DNA-binding transcription factors and other BAF subunits [44, 46].

The BAF60a is a tissue-specific role of the BAF60 isoforms, perhaps by association with distinct regulators. It is involved in the regulation of a specific metabolic gene network in the liver. Gatfield demonstrated that miR-122, which is a highly abundant, hepatocyte-specific microRNA, controls the circadian expression of BAF60a and mediates the regulation of cholesterol and lipid metabolism through a crosstalk with BAF60a and PPAR β/δ [47]. BAF60a is a diet-sensitive factor that controls a hepatic gene program responsible for bile acid synthesis and intestinal cholesterol absorption through a BAF60a/constitutive androstane receptor (CAR) of the feed-forward regulatory loop in the liver [48]. BAF60a activates a CAR-dependent program of gene expression in the liver to regulate bile acid and cholesterol metabolism. Disruption of this pathway by liver-specific inactivation of BAF60a protects mice from diet-induced hypercholesterolemia and atherosclerosis. The positive effects of BAF60a on glucose levels may due to the induction of gluconeogenic genes such as glucose-6-phosphatase (G6Pase) and phosphoenolpyruvate carboxykinase (PEPCK) and the accelerated rate of gluconeogenic process [46]. BAF60a may be also involved in fatty acid β-oxidation by interacting with PGC-1α [17]. Fatty acid β-oxidation is an important component of hepatic lipid homeostasis.

### BAF60c function in chromatin-remodeling

BAF60c, a component of the SWI/SNF chromatin-remodeling complex, is one of the first SWI/SNF subunits described to directly interact with transcriptional activators [49]. It is localized primarily in the cell nucleus and is expressed in a wide variety of tissues. BAF60c has been proposed to serve as a bridge between transcription factors and the ATPase-dependent chromatin remodeling BAF complex. BAF60c may play a role in heart development. BAF60c overexpressed in cell culture can mediate interactions between cardiac transcription factors and the BAF complex ATPase BRG1, thereby potentiating the activation of target genes [24, 50]. Silencing of BAF60c gene using siRNA in mouse embryos causes defects in heart morphogenesis as well as abnormal cardiac and skeletal muscle differentiation [24]. Studies found that BAF60c can regulate gene expression in muscle by interacting with MyoD [51, 52]. Recently, Baf60c has been identified that interacts with selective transcription factors, as a core component of a regulatory cascade that drives glycolytic myofiber formation [44]. It promotes a shift from oxidative to glycolytic metabolism in the muscle through its induction of Deptor expression and Akt activation [43, 44]. The oxidative-to-glycolytic metabolic shift was accompanied by higher glycolytic and lower oxidative gene expression. In addition, BAF60c is required for maintaining glycolytic capacity in adult skeletal muscle in vivo. BAF60c interacts with peroxisome proliferator-activated receptor γ (PPARγ) in a ligand-independent manner to enhance its transcriptional activity. The PPARγ is one of the three PPARs that together constitute a distinct subfamily of nuclear receptors. It has mostly been studied because of its key role in adipocyte differentiation, but it has many additional functions [16]. Nevertheless, Debril [49] reported that BAF60c does not seem to affect adipocyte differentiation and cell proliferation. On the other hand, BAF60c is specific for chromatin remodeling and transcription of lipogenic genes in response to insulin. BAF60c is reported to promote lipogenesis in vivo and that it increases triglyceride levels, demonstrating its role in metabolic adaption to activate the lipogenic program in response to feeding and insulin [18]. BAF60c is a subunit of functional diversity and may be related to the regulation of diseases.

## Hepatocyte heterogeneity and lipid metabolism

### Metabolic zonal functions in liver

The liver plays a most important metabolic role throughout the body, receiving its supply of hydrophilic nutrients absorbed by the intestine via the portal vein and delivering metabolized products to the other organs via the central vein [53]. Since the pioneering work of Jungermann on this subject, this functional heterogeneity is referred to as “metabolic zonation” of the liver, and demonstrated that the hepatocytes heterogeneity along the porto-central axis with respect to ultrastructure and enzyme activities resulting in different cellular functions within different zones of the liver lobuli [10, 54, 55]. Based on the location of the blood vessels, the terminal branches of the portal and the hepatic (central) veins and on the direction of the blood flow, hepatocytes of each liver lobule can be divided into two subpopulations, an upstream ‘periportal’ and a downstream ‘perivenous’ population [11]. Hepatocytes located in both zones of the liver lobuli show remarkable differences in the levels and activities of various enzymes and subcellular structures and thus have different metabolic capacities. The results obtained in several studies are summarized in Table 1. It is observed that gluconeogenesis, aminoacid degradation, ureagenesis, fatty-acid oxidation, and cholesterol synthesis are mostly located in the periportal hepatocytes whereas glycolysis, ketogenesis, glutamine synthesis, lipogenesis, and xenobiotic metabolism are preferentially situated in the perivenous hepatocytes [55, 62–65]. Therefore, the model of metabolic zonation proposes a functional specialization for the two zones of the liver.

**Table 1:** Predominant localization of the major metabolic functions and proteins in zones of hepatocytes

Periportal zone		Perivenous zone		References
Metabolic function	Protein	Metabolic function	Protein	
Lipid metabolism				
Fatty-acid oxidation, Cholesterol synthesis	3-hydroxyacyl-CoA dehydrogenase, CPI	Lipogenesis	ACC, ACL, FAS,	[10–12, 14, 15, 56, 57]
		Ketogenesis	β-hydroxybutyrate dehydrogenase,	
		Bile acid synthesis		
Glucose metabolism				
Gluconeogenesis	G6Pas, FBPas, PEPCK, Lactate dehydrogenase	Glycolysis	GK, PK_L_	[10, 11, 55, 58],
Glycogen synthesis				
	Alanine aminotransferase			
Ammonia and aminoacid utilization				
Urea synthesis, Aminoacid degradation	CPS1, OTC, ASS, Arg, TAT, SerDH, Alanine aminotransferase	Glutamine synthesis	GS	[10, 11, 55, 59–61]
	Aspartate aminotranferase			
Xenobiotic metabolism				
		Monooxygenation, Glucuronidation	Cytochrome P450 monooxygenases	[11, 55]
			GST, sulfotransferases	

*Abbreviations*: *CPI* carnitine palmitoyltransferase I, *ACC* acetyl-CoA carboxylase, *ACL* ATP citrate lyase, *FAS* fatty acid synthase, *G6Pas* glucose-6-phosphatase, *FBPas* fructose-1,6-bisphosphatase, *PEPCK* phosphoenolpyruvate carboxykinase, *GK* glucokinase, *PK*_*L*_ pyruvate kinase isoenzyme L, *CPS1* carbamoylphosphate synthetase, *OTC* ornithine carbamoyl transferas, *ASS* arginine succinate synthetase, *Arg1* Arginase 1, *TAT* tyrosine aminotransferase, *SerDH* serine dehydratase, *GS* glutamine synthase, *GST* glutathione S-transferase

### Periportal hepatocytes and fatty acid oxidation

Fatty acids are oxidized in three cellular organelles of liver cells, with β-oxidation confined to mitochondria and peroxisomes, with CYP4A catalyzed ω-oxidation taking place in the endoplasmic reticulum [66, 67]. The major pathway for the catabolism of fatty acids is mitochondrial fatty acid β-oxidation [66, 68]. This oxidation is a complex process taking place the in liver and involves the participation of several enzymes that are influenced by multiple factors. Enzymes such as carnitine palmitoyltransferase 1 (CPT1) and carnitine palmitoyltransferase 2 (CPT2), and substrates (fatty acid metabolism intermediates), such as malonyl-CoA are known to influence mitochondrial fatty acid β-oxidation [69]. The enzymes involved in the oxidation of fatty acids are also under a high degree of transcriptional control, and conditions that upregulate fatty acid β-oxidation are often associated with increases in the expression of a number of β-oxidation enzymes [70].

Fatty acid oxidation is a metabolic pathway underlying zonal expression in liver as two genes, encoding phosphatide phosphatase and apolipoprotein C2, a cofactor for activation of lipoprotein lipase, found in our study to be preferentially expressed in the periportal hepatocytes subpopulation [11]. In liver, fatty acids are utilized for oxidative energy supply, ketogensis and for the synthesis and release of triglycerides as very low-density lipoproteins. Moreover, only the liver can convert fatty acids to ketone bodies, which are important alternative energy substrates for many tissues, including the brain. Since the oxidative capacity is higher in periportal hepatocytes, it was postulated that the fatty acid oxidation via β-oxidation should also be localized in this zone [55].

### Perivenous hepatocytes and lipogenesis

Lipogenesis encompasses fatty acid synthesis and their utilization for phospholipid and triglyceride generation. By catalyzing seven reactions in fatty acid synthesis acetyl-CoA is converted to malonyl-CoA, the rate-limiting step in the lipogenesis pathway, catalyzed mainly by ACC [71]; Successive molecules of malonyl-CoA, which serves as a two carbon donor, are added to the acetyl-CoA primer by a multifunctional enzyme complex, the fatty acid synthase [72]. Fatty acid synthase plays a central role in *de novo* lipogenesis [73]; palmitic acid (C16:0) is the predominant fatty acid generated by fatty acid synthase [74]. Palmitic acid is desaturated by stearoyl-CoA desaturase-1(SCD-1) to palmitoleic acid or elongated to yield stearic acid (C18:0). Furthermore, studies have reported that the rate of lipogenesis and the activity of ACL, ACC, and FAS are considerably higher in the periportal than in the perivenous zone of the liver [12, 75].

In addition, fatty acid and fat synthesis in the liver is a highly regulated metabolic pathway that is important for very low-density lipoprotein (VLDL) production and thus energy distribution to other tissues. Docosahexaenoic and eicosapentaenoic acids reduced the secretion of chylomicron and VLDL partly by regulating the synthesis of triacylglycerol and apolipoprotein B [76]. Having common features at their promoter regions, lipogenic genes are coordinately regulated at the transcriptional level. Transcription factors, such as USFs, SREBP-1c, liver X receptors (LXRs) and carbohydrate-responsive element-binding protein (ChREBP) have crucial roles in this process. SREBPs are the basic helixloophelix leucine zipper (bHLH-LZ) transcription factors that bind as dimers to sterol regulatory elements (SREs) of target genes involved in lipid metabolism [77]. There are three SREBP isoforms: SREBP-1a, SREBP-1c, and SREBP-2, of which SREBP-1c is primarily responsible for the expression of lipogenic genes, although there is some functional overlap between different SREBPs. SREBP-1c is highly expressed in lipogenic tissue. It activates hepatic fatty acid synthesis through regulation of lipogenic genes [78, 79], such as ACL, ACC and FAS [80, 81]. SREBP-1c target genes encode a rate-limiting enzyme of the fatty acid elongase complex, which converts palmitate to stearate (C18:0) [80, 82]; the stearoyl-CoA desaturase-1, which converts stearate to oleate (C18:1); and glycerol-3-phosphate acyltransferase, the first committed enzyme in triglyceride and phospholipid synthesis [80, 83]. In addition to SREBP-1c, the critical role of USF in lipogenic gene transcription has been demonstrated in vivo in USF-knockout mice that have significantly impaired lipogenic gene induction [84]. Although it is possible that other SREBP isoforms might compensate for SREBP-1c, the partial effect of its ablation on hepatic lipogenesis is more likely to reflect the contribution of other transcription factor(s), such as USFs, ChREBP, and LXRs, which are required for, lipogenic gene induction or its enhancement. Having common features at their promoter regions, lipogenic genes are coordinately regulated at the transcriptional level.

## Nutrient supply and the regulation of BAF60a on hepatic fatty acid oxidation

### Regulation of fat oxidation by PGC-1α and PPARα

Fatty acid oxidation occurs in the liver and is strongly enhanced during fasting as a result of increased fatty acid influx and altered hormonal signals. The increase of fat oxidation during fasting is accompanied by transcriptional activation of peroxisomal and mitochondrial fatty acid β-oxidation genes. The gene program of fat oxidation is regulated by several transcription factors and cofactors, including PPARα, PGC-1α, and BAF60a [17, 85]. Studies have shown that PPARα plays a key role in he transcriptional control of encoding genes related to lipid metabolism in the liver, including those involved in mitochondrial β-oxidation, peroxisomal β-oxidation, fatty acid uptake and/or binding, and lipoprotein assembly and transport [86–89]. PPARα is a member of the nuclear hormone receptor superfamily. It is primarily expressed in brown adipose tissue and the liver, and to a lesser extent in the kidneys, skeletal muscle, and heart [90]. Previous studies have demonstrated that PPARα transcriptionally by regulates all key enzymes of peroxisomal and mitochondrial β-oxidation pathways [66, 91–96]. PPARα-null mice were either fasted or fed a high fat diet to investigate the role of PPARα under these physiological conditions [97]. Fasted PPARα-null mice show enhanced accumulation of lipid in the liver, suffer from severe hypoglycemia and hypothermia, and reveal a dramatic inhibition of fatty acid uptake and oxidation. Furthermore, it is shown that to accommodate the increased requirement for hepatic fatty acid oxidation PPARα mRNA is induced in wild-type mice during fasting. These results indicate that PPARα is a key transcriptional regulator of fatty acid oxidation genes and is essential for hepatic fat oxidation during starvation.

In addition, studies have also found that PGC-1α activates the expression of PPARα target genes involved in hepatic fatty acid oxidation [98, 99]. PGC-1α physically interacts with PPARα and increases its transcriptional activity [100]. One of the roles for PGC-1 as a PPARα co-activator is to control cellular fatty acid oxidation [100]. PGC-1 is critical for PPARα interaction and transcriptional activation, two distinct processes. PGC-1α has been identified as a binding partner and co-activator of the transcriptional activity of PPARγ as part of a study to identify transcriptional components of the pathway responsible for metabolic changes that include glucose and lipid homeostasis and mitochondrial oxidative metabolism [101]. It in enriched in metabolic tissues such as muscle, heart, and liver, where it interacts with multiple DNA-binding transcription factors. The co-transcriptional activity of PGC-1α in liver is important for the compensatory metabolic responses that occur during food deprivation. Acute RNA interference-mediated PGC-1α knockdown leads to profound down-regulation of fatty acid oxidation gene expression during short-term starvation [98]. Rates of fatty acid oxidation are also diminished in isolated hepatocytes from PGC-1α-deficient mice [102]. In this complex hormonal and nutrient regulation, PGC-1α is controlled and recruited to regulate gene expression of hepatic fatty acid oxidation enzymes. Studies shown that general control non-repressed protein 5 (GCN5) is able to acetylate PGC-1α and suppress its activity, but evidence for the dietary control of this process is somewhat limited [103]. In contrast, PGC-1α is heavily deacetylated by Sirtuin-1 NAD^+^-dependent deacetylase (SIRT1) and activates its activity. SIRT1 is a NAD^+^-dependent protein deacetylase that has been implicated in several physiological processes in mammals, including control of lipolytic rates in white adipose tissue. Moreover, levels of NAD^+^ have been shown to change in response to nutrient availability. For example, the concentration of intracellular NAD^+^ increased during fasting in the liver of rodents [87, 104, 105]. Consequently, acetyl transferase GCN5 and deacetylase SIRT1 are capable of changing the acetylation state of PGC-1α in response to nutrient state and reciprocally alter the transcriptional coactivating properties of PGC-1α [106, 107], providing a new metabolic regulator that allows mammalian cells to switch from glucose to fatty acid oxidation in nutrient deprivation conditions [108]. Recently, the hormone fibroblast growth factor (FGF21) was also shown to be induced in liver during fasting, induces hepatic expression of PGC-1α, and activation of hepatic lipid oxidation, triglyceride clearance, and ketogenesis [109–111]. PGC-1α participates in the regulation of fatty acid oxidation by a complex network of transcription factors under the condition of hormonal and nutrient signals.

### Interaction of BAF60a, PGC-1α, and PPARα

BAF60a is a subunit of the SWI/SNF chromatin-remodeling complexes that activates or represses the transcription of diverse target genes. Studies found that BAF60a induces the expression of genes involved in peroxisomal fatty acid β-oxidation in a dosedependent manner, including acetyl-Coenzyme A acyltransferase 1B (Acaa1b), acyl-Coenzyme A oxidase 1 (Acox1), hydroxyacyl-CoA dehydrogenase (Hadha), and enoyl-Coenzyme A hydratase 1 (Ech1) [17]. BAF60a also induces the expression of mitochondrial fatty acid β-oxidation genes, such as acetyl-CoA acyltransferase 2 (Acaa2), CPT1, and Hadha [17]. Also in that study in mice liver, the RNAi knockdown RNAi knockdown of BAF60a were studied and showed that the mRNA levels of key enzymes in the fatty acid β-oxidation pathway, including Acaa1b, Acox1, Acaa2, and Hadha are significantly lower. These results suggest BAF60a plays an important role in hepatic fat oxidation.

BAF60a as a transcription factor has been demonstrated to link the SWI/SNF complexes to the transcriptional coactivator PGC-1α, suggesting there is a role for BAF60a in hepatic lipid metabolism. Adenoviral-mediated expression of BAF60a stimulates the entire program of peroxisomal and mitochondrial fat oxidation and lowers liver triglyceride content in mouse models of hepatic steatosis. Also, BAF60a is required for the activation of hepatic fat oxidation during fasting. PGC-1α was found to be essential for the function of BAF60a as a regulator of fatty acid oxidation genes to transcriptional activation of peroxisomal and mitochondrial lipid oxidation genes in hepatocytes. Since both BAF60a and PGC-1α increase mRNA levels of several fatty acid β-oxidation genes, including Acaa1b, Acox1, and Hadha simultaneous expression of these two factors leads to significantly higher induction of target genes [7].

BAF60a is a recently described circadian regulator that links time of day to liver metabolic physiology. Tao demonstrated that BAF60a plays a critical role in the coordinated regulation of hepatic circadian clock and energy metabolism in mammals [46]. Knockdown of BAF60a in the liver significantly disrupted the rhythmic expression patterns of clock genes including Bmal1, Per1, Per2, Rev-erbα, and Cry1, as well as of genes involved in key metabolic pathways including gluconeogenesis, glucose oxidation, fatty acid β-oxidation, and mitochondrial respiration. Other studies found that BAF60a is also an important integrator linking the circadian clock and the physiological homeostasis of vascular smooth muscle cells (VSMCs) [112]. BAF60a co-activates with the retinoid-related orphan receptor (ROR) family of orphan nuclear receptors to stimulate Bmal1 transcription and is essential for normal circadian rhythms in VSMCs. Notably, during the development of cardiovascular diseases, such as atherosclerosis, the phenotype of VSMCs will transit from contractile to synthetic and, correspondingly, there are noticeable cell behavior changes [113]. Similarly, PGC-1α is a critical component of the mammalian clock. PGC-1α stimulates the expression of Bmal1 through coactivating the ROR family of orphan nuclear receptors and is essential for normal circadian rhythms [114].

Moreover, BAF60a and PPARα have a functional crosstalk in the regulation of fatty acid oxidation gene transcription [17]. Previous transcriptional profiling studies have demonstrated that activation of PPARα by WY14643 (an experimental hypolipidemic drug) was found to increase the induction of fatty acid oxidation genes (Acaa1b, Acox1, and Hadha) by BAF60a providing more evidence for the existence of a connection between BAF60a and the PPARα pathway [17, 115]. The transcriptional function of BAF60a is significantly impaired in PPARα-null hepatocytes. Despite this, BAF60a is still capable of activating the expression of fatty acid oxidation genes in the absence of PPARα, suggesting that both PPARα-dependent and PPARα-independent pathways mediate the metabolic effects of BAF60a on hepatic fat oxidation [17]. At the molecular level, BAF60a is required to PPARα-binding sites on the fatty acid oxidation gene. BAF60a may be recruited for fatty acid oxidation gene promoters through its direct interaction with PPARα [17]. PPARα plays a pivotal role in the control of cellular fatty acid utilization pathways in response to diverse physiologic conditions including fasting [97, 116, 117], nutritional alterations [97], and aging [118]. The PPARα regulatory pathway has also been implicated in disease states including cardiac hypertrophy [119], obesity [120], and diabetes mellitus [116, 120, 121]. The BAF60a/PGC-1α complex is required for the transcriptional function of PPARα in the context of hepatic lipid metabolism. BAF60a also induces the expression of PEPCK as well as several clock genes, raising the possibility that the BAF60a/PGC-1α interaction may play a more general role in the transcriptional regulation by PGC-1α [122]. The interaction of BAF60a with PGC-1α and PPARα, leads to stimulation of specific transcriptional programs.

## Nutrient supplies and the regulation of BAF60c on hepatic lipogenesis

### The physical interaction between BAF60c and USF

USF proteins are members of the basic-helix-loop-helix (bHLH) family of transcription factors. First identified for their involvement in transcription from the adenovirus major late promoter [123, 124], USF proteins were purified as the 43-kDa USF-1 and 44-kDa USF-2 [121]. USF-1 and USF-2 are homo-or heterodimers that bind to an E-box with identical DNA binding specificity as target promoters for transcriptional activation [125, 126]. USFs are found to bind to the proximal promoter region of the gene encoding fatty acid synthase, which is a key enzyme in lipogenesis [126]. It has been shown that USFs are required for FAS promoter activation by insulin by binding to the -65E-box [127]. The critical role of USF in lipogenic gene transcription has been demonstrated in vivo using USF knockout mice that have significantly impaired lipogenic gene induction [84]. Together, these results indicate that phosphorylation-dependent acetylation of USF-1 functions as a sensor for nutritional/insulin status to activate FAS transcription. Many other lipogenic genes contain closely spaced E-boxes and SREs in their proximal promoter regions, and therefore may also be subject to transcriptional regulation by USF-1. USF plays a pivotal role as a molecular switch by recruiting distinct transcription factors and coregulators in a fasting/feeding- dependent manner [128].

Since BAF60 is known to function as an anchor point between transcription factors and the BAF complex, Wong [5] first found that BAF60c directly interacts with USF-1 to form lipogenic gene promoters upon feeding. After feeding, insulin levels rise, which activates lipogenic genes through several pathways, including the DNA-dependent protein kinase (DNA-PK), atypical protein kinase C (aPKC) and AKT-mTOR pathways, as well as protein phosphatases such as protein phosphatase 1 (PP1) and protein phosphatase 2 (PP2) [128, 129]. These pathways control the post-translational modifications of transcription factors and co-regulators, such as phosphorylation, acetylation or ubiquitylation that affect their function, stability and/or localization. In a study BAF60c was identified as USF-interacting protein and found that BAF60c was phosphorylated by aPKC upon feeding/insulin [5]. In response to insulin/feeding, BAF60c was phosphorylated by aPKC, causing translocation of BAF60c to the nucleus and allowing a direct interaction of BAF60c with USF-1 that is phosphorylated by DNA-PK and acetylated by P/CAF. This BAF60c phosphorylation, together with USF acetylation is required for the interaction between two proteins. Furthermore, Overexpression of BAF60c activated the lipogenic transcription program in mice even in the fasted state. BAF60c appears to serve as the anchor point bridging USF-1 and BAF complex for lipogenic gene transcription. Since BAF60c is the specific isoform for lipogenic gene activation, defined the USF-1 interacts with BAF60c recruits BAF subunits, including BAF155, BAF190, and BAF250 for the formation of the lipoBAF for lipogenic gene transcription, which is based on phosphorylation dependent translocation of BAF60c. Therefore, USF acetylation and BAF60c phosphorylation are required for the interaction between both in converging insulin signals [51].

### BAF60c induces lipogenesis in feeding/insulin

The liver is the organ responsible for the conversion of excess carbohydrates to fatty acids to be stored as triglycerides or burned in muscle. A classic action of insulin is to stimulate fatty acid synthesis in liver during times of carbohydrate excess. Lipogenesis in liver is under nutritional and hormonal control. Enzymes involved in these processes are tightly and coordinately regulated during fasting and feeding/insulin at the transcriptional level. It should be noted that insulin and glucagon also exert a posttranslational control of fatty acid synthesis through changes in the phosphorylation and activation of acetyl-CoA carboxylase. The transcription of these enzymes is low in fasting, whereas a high carbohydrate meal that raises insulin levels activates the lipogenic transcription program [126, 130, 131]. Insulin-mediated activation of atypical PKCζ/λ via the PI3K pathway induces SREBP-1c expression and lipogenesis [132–136].

BAF60c is phosphorylated by aPKC, which causes translocation of BAF60c to the nucleus and allows a direct interaction of BAF60c with phosphorylated/acetylated USF. This phosphorylation, together with USF acetylation are required for the interaction between two proteins. BAF60c forms the lipoBAF complex for chromatin remodeling that is required for activation of the lipogenic program. BAF60c overexpression activates the lipogenic program by increasing expression of a variety of lipogenic enzymes, including FAS, ACC, ACL, stearoyl-CoA desaturase-1(SCD1) [18]. Thus, forced overexpression of BAF60c enhanced lipogenesis in vivo even in the fasted condition when no significant *de novo* lipogenesis is expected to normally occur. Overall, BAF60c plays important roles in chromatin remodeling and transcription of lipogenic genes in response to insulin.

Multiple lines of evidence suggest that the stimulatory effect of insulin on fatty acid synthesis is mediated by an increase in SREBP-1c. The elevated SREBP-1c increases lipogenic gene expression, enhances fatty acid synthesis, and accelerates triglyceride accumulation [137, 138]. The transcription of SREBP-1c is regulated by three factors selectively: LXRs, insulin, and glucagon. One study found that the bHLH domain of USF directly interacts with the bHLH and an N-terminal region of SREBP-1c for their synergistic activation of the promoter [6]. The closely spaced arrangement of the E-box and SRE in many lipogenic promoters could allow USF and SREBP-1c to cooperatively activate lipogenic gene transcription [6]. Co-immunoprecipitation studies using USF-1 mutants containing S262D and S262A showed that S262 phosphorylation increases its interaction with SREBP-1c [128]. Another study found that SREBP-1c binding to SRE is USF dependent, as SREBP-1c could not bind SRE in the FAS promoter when the nearby E-box was mutated [139]. The requirement of USFs for SREBP-1c function was revealed by USF knockout mice that showed severely delayed FAS induction during feeding, even when the level of the mature form of the SREBP remained unchanged [84]. In addition, SREBP-1, which itself is induced upon feeding/insulin treatment is involved in the activation of lipogenic genes [79], was also identified to be activated upon BAF60c overexpression. Thus, USF bound to the -65 E-box recruits SREBP-1c to bind the nearby SRE during feeding/insulin Furthermore, functional domain mapping using USF1- and SREBP1c-deletion constructs indicated that the activation domains of both proteins are required for this functional synergy. This indicates that post-translational modifications of USF-1 are crucial for the recruitment of SREBP-1c to bind to the nearby SRE for the synergistic activation of lipogenic genes by USFs and SREBP-1c during feeding or insulin treatment [128]. Taken together, identification of SREBP-1c, BAF60c as USF interacting proteins has led to the discovery of novel players in insulin signaling cascade and has revealed an unexpected link between DNA break/repair and metabolism.

## Conclusion

The liver plays a considerable role in the homeostasis of lipid metabolism. It regulates several major aspects of lipid metabolism, including lipogenesis, lipoprotein uptake and secretion, and fatty acid β-oxidation. Fatty acids serve as an important source of energy as well as energy storage for many organisms and are also pivotal for a variety of biological processes, including the synthesis of cellular membrane lipids and generation of lipid-containing messengers involved in signal transduction [140]. Fatty acids can generally be stored efficiently as non-toxic triglycerids. Accordingly, the regulation of lipid metabolism is critical since deregulated lipogenesis and fatty acid oxidation physiological processes are often linked to pathological conditions including hepatic steatosis, diabetes, and cardiovascular disease. Recent studies have been implicated in hepatic steatosis, occurring when the balance of triglyceride is disrupted, due to increased *de novo* lipogenesis and fatty acid uptake and reduced fatty acid oxidation and very low-density lipoprotein [141, 142], but the molecular is still poorly understood. Recently, studies proposed that n-3 PUFA is a qualified nutritional regimen fighting against metabolic disorders [143]. Thus, the regulation of lipid metabolism may be targeted to therapeutic hepatic steatosis.

**Fig. 2:**
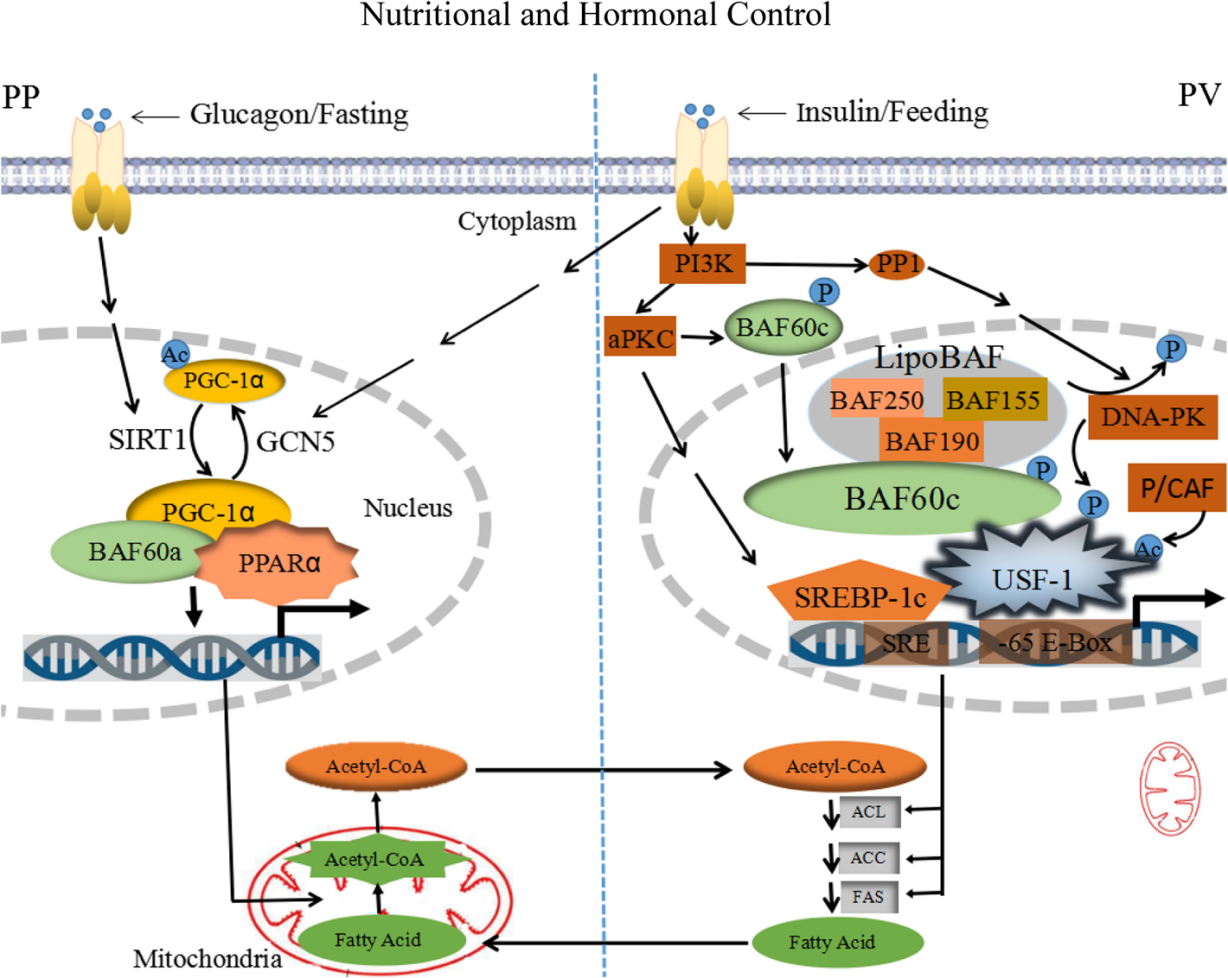
Regulation of lipid metabolism by BAF60a and BAF60c in periportal and perivenous hepatocytes, respectively, under the control of nutritional and hormonal signals. In periportal (PP) hepatocytes, PGC-1α is deacetylated by SIRT1 and activates its activity during fasting. In this state, PGC-1α mediates the recruitment of BAF60a to PPARα-binding sites, to transcriptional activation of mitochondrial fat-oxidation genes, leading promoting the oxidation of fatty acids. The acetyl-CoA is produced by fatty acid oxidation and transported from mitochondria to cytoplasm. In perivenous (PV) hepatocytes, insulin-mediated activation of atypical PKCζ/λ via the PI3K pathway induces SREBP-1c expression. BAF60c recruits BAF subunits including BAF155, BAF190, and BAF250 for the formation of lipoBAF complex to activate lipogenic program. BAF60c is phosporylated by aPKC in response to feeding/insulin. Phosphorylated BAF60c translocates from the cytosol to the nucleus and directly interacts with phosphorylated/acetylated USF, thus allowing recruitment of lipoBAF and remodeling of chromatin to activate lipogenic genes. USF-1, which is phosphorylated by DNA-PK and then acetylated by P/CAF, recruits BAF60c. DNA-PK is activated by PP1. USF-1 bound to the -65 E-box recruits SREBP-1c to bind the nearby SRE during feeding/insulin. The closely spaced arrangement of the E-box and SRE in many lipogenic promoters may allow USF-1 and SREBP-1c to cooperatively activate lipogenic genes transcription, leading to increased in the expression of ATP citrate lyase (ACL), acetyl-CoA carboxylase (ACC), and fatty acid synthase (FAS), to facilitate the synthesis of fatty acids. Moreover, PGC-1α is acetylated by GCN5 resulting in a transcriptionally inactive protein in response to feeding/insulin. Since fatty acid oxidation occur mainly in PV hepatocyte, whereas lipogenesis occur predominantly in PP hepatocyte. Therefore, the acetyl-CoA in PP hepatocyte is transported into PV hepatocyte and used for fatty acid synthesis, whereas the fatty acid in PV hepatocyte is shifted to PP hepatocyte and oxidated to acetyl-CoA

However, lipid metabolism is mediated by transcription factors BAF60a and BAF60c, all of which are positive modulators of hepatic triglyceride contents by targeting genes coding for key reactions in lipid metabolism. The recruitment of BAF60a is mediated by PGC-1α to PPARa-binding sites, leading to transcriptional activation of hepatic fatty acid oxidation genes, as discussed above. Phosphorylation and recruitment of BAF60c promotes lipogenesis and increases triglyceride levels in response to feeding and insulin. Thus, BAF60a and BAF60c define a critical link between the SWI/SNF chromatin-remodeling complexes and hepatic lipid metabolism. Additionally, the SWI/SNF chromatin-remodeling complexes play important roles in the regulation of hepatic lipid metabolism. Interestingly, previous studies demonstrated the expected heterogeneity in levels between periportal and perivenous hepatocytes, for example, the activities of various enzymes and metabolic functions. Remarkably, lipid metabolism is also zoned in these two hepatic regions. Fatty acid oxidation is exclusively active periportal hepatocytes, whereas lipogenesis is exclusively active perivenous hepatocytes. Zonal-specific expression has also been established for key enzymes of lipid metabolism, showing, for example, a higher activity of 3-hydroxyacyl-CoA dehydrogenase in periportal cells compared to perivenous hepatocytes. In a word, under the control of nutritional and hormonal signals, lipid metabolism is regulated through BAF60a and BAF60c in periportal and perivenous hepatocytes, respectively (Fig. 2). Therefore, functions and expression of BAF60a and BAF60c may be associated with hepatocyte heterogeneity. Whether other subunits of the SWI/SNF chromatin-remodeling complexes have the zonal hepatocyte-characteristic heterogeneity that is responsible for the regulation of metabolic differences.

There is increasing evidence that alterations in chromatin remodeling play a significant role in human disease. Alteration of DNA-histone contacts within a nucleosome in an ATP-dependent manner changes the chromatin structure. The SWI/SNF complex is an ATP-dependent chromatin-remodeling complex. Subunits of SWI/SNF complexes have recently been implicated as tumor suppressors in human malignancies [144]. It primarily disorganizes and reorganizes nucleosome positioning to promote accessibility for transcription-factor binding and gene activation, and regulates both transcriptional activation and repression. It also plays an important role in regulation of lipid metabolism during different nutritional and hormonal conditions, as the SWI/SNF complex might be targeted to develop drugs aimed at regulation of lipid homeostasis in hepatic steatosis. In this review, we present novel insight into therapeutics of hepatic steatosis through the SWI/SNF chromatin-remodeling complexes regulates lipid homeostasis.

## Abbreviations

Acaa1b: acetyl-Coenzyme A acyltransferase 1B
Acaa2: acetyl-CoA acyltransferase 2
ACC: acetyl-CoA carboxylase
ACL: ATP citrate lyase
Acox1: acyl-Coenzyme A oxidase 1
aPKC: atypical protein kinase C
BAFs: BRG1- or BRM-associated factors
bHLH: basic-helix-loop-helix
BRG1: brahma-related gene 1
BRM: brahma
CHD: chromodomain and helicase-like domain
ChREBP: carbohydrate-responsive element-binding protein
CPT1: carnitine palmitoyltransferase 1
CPT2: carnitine palmitoyltransferase 2
DNA-PK: DNA dependent protein kinase
Ech1: enoyl-Coenzyme A hydratase 1
FAO: fatty acid β-oxidation
FAS: fatty acid synthase
FGF21: fibroblast growth factor
GCN5: general control non-repressed protein 5
Hadha: hydroxyacyl-CoA dehydrogenase
INO80: inositol requiring 80
ISWI: imitation SWI
LXR: liver X receptor
PGC-1α: peroxisome proliferator-activated receptor γ coactivator protein-1α
PP1: protein phosphatases 1
PPARα: peroxisome proliferatoractivated receptor-α
SCD1: stearoyl-CoA desaturase-1
SIRT1: sirtuin-1 NAD^+^-dependent deacetylase
SREBP-1c: sterol regulatory elementbinding protein 1c
SREs: sterol regulatory elements
SWI/SNF: switching defective/sucrose non-fermenting
USF: upstream stimulatory factor
VLDL: very low-density lipoprotein
